# Prognostic and Therapeutic Significance of Cancer‐Associated Fibroblasts Genes in Osteosarcoma Based on Bulk and Single‐Cell RNA Sequencing Data

**DOI:** 10.1111/jcmm.70424

**Published:** 2025-03-05

**Authors:** Yukang Que, Tianming Ding, Huming Wang, Shenglin Xu, Peng He, Qiling Shen, Kun Cao, Yang Luo, Yong Hu

**Affiliations:** ^1^ Department of Orthopedics The First Affiliated Hospital Anhui Medical University Hefei Anhui China; ^2^ Department of Orthopedics Yangzhou East Hospital Yangzhou Jiangsu China

**Keywords:** cancer‐associated fibroblasts, genes, osteosarcoma, prognosis, single‐cell RNA sequencing

## Abstract

Osteosarcoma (OS) is the most frequent primary solid malignancy of bone, whose course is usually dismal without efficient treatments. The aim of the study was to discover novel risk models to more accurately predict and improve the prognosis of patients with osteosarcoma. The single‐cell RNA sequencing (scRNA‐seq) data was obtained from the GEO database. Bulk RNA‐seq data and microarray data of OS were obtained from the TARGET and GEO databases respectively. A clustering tree was plotted to classify all cells into different clusters. The “cellchat” R package was used to establish and visualise cell–cell interaction networks. Then Univariate COX regression analysis was used to determine the prognostic CAF‐related genes, followed by the Lasso‐Cox regression analysis to build a risk on the prognostic CAF‐related genes. Finally, from multiple perspectives, the signature was validated as an accurate and dependable tool in predicting the prognosis and guiding treatment therapies in OS patients. From the single‐cell dataset, six OS patients and 46,544 cells were enrolled. All cells were classified into 22 clusters, and the clusters were annotated to 14 types of cells. Subsequently, CAFs were observed as a vital TME components. In cell–cell interaction networks in OS cells, CAFs had a profound impact as four roles. Via the Univariate COX regression analysis, 14 CAF‐related genes were screened out. By the Lasso‐Cox regression analyses, 11 key CAF‐related genes were obtained, based on which an 11‐gene signature that could predict the prognosis of osteosarcoma patients was constructed. According to the median of risk scores, all patients were grouped in to the high‐ and low‐risk group, and their overall survival, activated pathways, immune cell infiltrations, and drug sensitivity were significantly differential, which may have important implications for the clinical treatment of patients with osteosarcoma. Our study, a systematic analysis of gene and regulatory genes, has proven that CAF‐related genes had excellent diagnostic and prognostic capabilities in OS, and it may reshape the TME in OS. The novel CAF‐related risk signature can effectively predict the prognosis of OS and provide new strategies for cancer treatment.

**Abbreviations**
AUC: area under the curve
CAFRG: CAF‐related gene
CAFs: cancer‐associated fibroblasts
CYT: Cytolytic cytolytic activity
GDSC: Drug drug Sensitivity sensitivity in Cancercancer
GEO: Gene gene Expression expression Omnibusomnibus
GO: Gene gene Ontologyontology
GSEA: Gene gene Set set Enrichment enrichment Analysisanalysis
HRs: Hazard hazard ratios
IC50: the half maximalhalf‐maximal inhibitory concentration
KEGG: Kyoto Encyclopedia of Genes and Genomes
KM: Kaplan–Meier
MSigDB: Molecular molecular Signatures signatures Databasedatabase
NMF: Nonnon‐negative Matrix matrix Factorizationfactorization
OS: osteosarcoma
PCA: principal component analysis
ROC: Receiver receiver operating characteristic
scRNA‐seq: signal‐cell RNA sequencing
TARGET: Therapeutically therapeutically Applicable applicable Research research to Generate generate Effective effective Treatmentstreatments
TLS: Tertiary tertiary lymphoid structure
TME: tumourtumor microenvironment
TOM: topological overlap matrix
TPM: transcripts per million
UMAP: uniform manifold approximation and projection
UMI: unique molecular identifiers
β: coefficient values

## 1 Introduction

Osteosarcoma (OS) is a metaphyseal malignant bone malignancy composed of mesenchymal cells producing osteoid and immature bone, and it may rarely happen among soft tissues [1]. Osteosarcoma is more prone to affect adolescence at 15–19 years of age, with an annual incidence of 8–11/million/year [2]. The tentative diagnosis of osteosarcoma can be deduced from radiograph results, but pathological evaluation of a bone biopsy specimen is necessary for definitive conclusions and CT scans of the chest should be performed to identify lung nodules [3]. The pathogenesis of osteosarcoma has been widely studied but is still not very explicit. Gene alterations including the loss of TP53 and other oncogenic events are commonly observed in osteosarcoma [4]. Due to the lack of effective therapies and the high heterogeneity, local and distant metastasis will lead to bad clinical outcomes, therefore it is devastating and a challenging issue worldwide. So, there is a necessity to explore novel and credible prognostic indicators containing more abundant genetically‐based information that can be used to lead to a more timely and rational process of disease detection, diagnosis, and prognosis prediction. To try to solve this issue, this study was conducted, to establish an accurate prediction signature for osteosarcoma patients based on the single‐cell and bulk RNA datasets to provide more instructions on disease prevention, recognition and control.

The tumour microenvironment (TME) includes a variety of immune cells, cancer‐associated fibroblasts (CAFs), endothelial cells, and a variety of other tissue receptor cells. The ongoing interactions between tumour cells and the TME play decisive roles in tumour initiation, progression, metastasis, and response to therapies, which has made the TME an important therapeutic target in cancer [5, 6]. The TME is highly heterogeneous. Therefore, TME‐mediated drug resistance is hard to avoid [7]. It can be induced by soluble factors secreted by tumour or stromal cells, or adhesions of tumour cells to stromal fibroblasts or components of the extracellular matrix [8]. Targeting the TME has revolutionised the keynote of cancer treatments. Responses to radiotherapy [9] or immunotherapy [5, 10] make improvements accordingly.

Among all TME cells, CAFs as a key element in the TME of solid tumours, have caught the attention because they are thought to have diverse functions, including matrix deposition and remodelling, extensive reciprocal signalling interactions with cancer cells and crosstalk with infiltrating leukocytes [11]. CAFs are known as accomplices in tumour malignancy [12, 13]. As a result, a promising way of targeting CAFs by altering their numbers, subtypes or functions, has emerged to enhance cancer therapy efficiency [14]. Further understanding of the TME and CAFs would be referentially valuable in providing personalised treatment strategy practically.

The single‐cell RNA sequencing (scRNA‐seq) technology allows the dissection of gene expression at single‐cell resolution, which has dramatically reformed transcriptomic researches [15]. In recent years, the scRNA‐seq technique has already been applied in the exploration of various cancers [16]. Moreover, in the single‐cell RNA‐sequencing era, the knowledge of CAF biological diversity has been extremely expanded regardless of their high heterogeneity [17, 18].

In this study, CAF‐related genes were identified based on scRNA‐seq datasets. Eventually, an 11‐gene signature was constructed and demonstrated to have satisfying accuracy in predicting the prognosis of osteosarcoma patients and the signature could be served as an independent prognostic factor. Therefore, the novel signature provided new ideas for exploring molecular mechanisms and designating targeted therapies of osteosarcoma.

## 2 Materials and Methods

### 2.1 Data Acquiring and Preprocessing

A scRNA‐seq dataset (accession number: GSE162454) including six patients and 46,544 cells was downloaded from the Gene Expression Omnibus (GEO; https://www.ncbi.nlm.nih.gov/geo/) database [19, 20]. Relevant clinical information and RNA‐seq data regarding osteosarcoma patients were obtained from the Therapeutically Applicable Research to Generate Effective Treatments (TARGET; https://ocg.cancer.gov/programs/target) database, with 93 samples included and set as the training dataset. From the GEO database, 47 samples (GSE21257) were acquired and set as the verification cohort. Via the “Combat” algorithm from the “sva” R package, the possible batch effects of abiotic bias in the two datasets were reduced [21].

### 2.2 Identifying Biomarkers of CAFs Based on the Single‐Cell Dataset

The “Seurat” R package was adopted to perform the analysis and realise quality control (minimum gene numbers > 500; maximum gene numbers < 4000; mitochondria gene percentage < 10%), thus avoiding low‐mass cell, zero‐cell or multicellular capture. The “SCTransform” function was used to standardise the data, and its rectifying effect on the depth of sequencing was better than that of the “log” standard. The “RunHarmony” function was used to integrate the single sample. Compared to other algorithms, the “Harmony” algorithm can integrate data while still being sensitive to rare cells, making it suitable for more complex single‐cell assay designs that can compare cells from different donors, tissues, and technology platforms [22, 23]. The principal component analysis (PCA) was conducted to reduce the dimensionality of high‐variable genes by the “RunPCA” function and the first 30 PCs were used. Via the “clustree” R package, 0.6 was chosen as the resolution. The “FindClusters” function was used to identify the clusters and the “RunUMAP” function was to visualise the clusters. Subsequently, the cell clusters were annotated manually according to cell‐specific lineage genes. Based on the “FindAllMarkers” function, genes that were highly expressed in each cell subpopulation were identified as markers for each cell group.

### 2.3 Performing Mechanism Analyzes at the Single‐Cell Level

Firstly, in every patient from the single‐cell dataset, the average number and proportion of 14 different cell types were compared. From the Molecular Signatures Database (MSigDB) [24, 25], the hallmark gene sets were acquired. Subsequently, the “irGSEA” and “UCell” [26] R packages were applied to evaluate gene signatures by scoring each pathway in the gene set. Next, we collected some proliferative genes (AURKA, BIRC5, CCNB1, CCNE1, CDC20, CDC6, CENPF, CEP55, EXO1, MKI67, KIF2C, MELK, MYBL2, NDC80, ORC6, PTTG1, RRM2, TYMS, UBE2C) and termed them as a new gene set. The UMAP visualised the distribution of the gene set score in the 14 cells. Then, using the “PROGENy” R package [27], PROGENy scores of the 14 cells in different signalling pathways were calculated. The activity of transcription factors (TFs) was inferred by the “DoRothEA” R package. Using the “cellchat” R package, the communicating interactions between 14 different cell subgroups (myeloid cells, CD8T cells, osteosarcoma cells, T cells, exhausted CD8 T cells, CAFs, exhausted T cells, NK cells, plasma cells, proliferative CD8 T cells, endothelial cell, osteoclasts, B cells, mast cell) was studied and the mechanism of the communicating molecules at the single‐cell resolution was identified. Interaction number and strength were shown. Then the cell communicating landscape was described to identify the communication mode of the TME in OS.

### 2.4 Explaining Complex Intercellular Communication Networks

Cell–cell interactions are essential for the information exchange between different cells and are important in laying the foundation for multiple biological processes. Continuing utilising the “cellchat” R package [28], the cell–cell networks were quantified from perspectives of graph theory, pattern recognition and multiple learning. The dominant transmitter (source) and receiver (target) were identified and visualised in 2D space. Then we analysed and visualised multiple ligand‐receptor mediated cell interactions for the 14 kinds of cells as mentioned before. After that, interactions between various osteosarcoma cells and receptors were analysed emphatically. Subsequently, the role (sender, receiver, mediator, or influencer) of each of the 14 cells in the MIF and MK signalling pathway has been identified by calculating the network centrality index of each cell subgroup. Next, using the “extractEnrichedLR” function, all important interactions (Ligand‐Receptor pairs) and associated signalling genes that contributed most to the MIF or MK signalling pathway have been identified. And, the Non‐negative Matrix Factorization (NMF) algorithm was applied to recognise cell communication patterns. Using cophenetic and silhouette measure types, outgoing and incoming signalling patterns were identified. After choosing the best pattern number in outgoing and incoming signalling, associations of certain patterns with different cell populations and ligand‐receptor pairs or signalling pathways were visualised.

### 2.5 Verifying That CAFs May Contribute to the Oncogenic Characteristics of Sarcomas Through the MIF‐CD74 Signalling Axis

To enhance the reliability of our results, we combined the normal data from the GTEx database to increase the sample size with the tumour data from TCGA. The differential analysis was conducted on MIF expression using the Wilcox. test function. The HPA database summarises each gene's transcript expression levels across 28 types of cancer. Protein–protein interaction data often include biologically unlikely interactions. Here, we utilised the comPPI database (https://comppi.linkgroup.hu/) to filter out protein interactions that do not share a common subcellular localization, ultimately obtaining proteins that interact with the target gene. To ensure the quality and consistency of the data, immune infiltration data for all TCGA samples were collected from the TIMER2.0 database. The CAF scores were estimated by the EPIC algorithm. Based on the mean value, patients were divided into four groups: high CAFs_EPIC infiltration and high MIF expression, low CAFs_EPIC infiltration and low MIF expression, high CAFs_EPIC infiltration and low MIF expression, and low CAFs_EPIC infiltration and high MIF expression. The log‐rank test was used to compare the two groups' survival differences pairwise. Likewise, patients were divided into four groups: high CD74 expression and high MIF expression, low CD74 expression and low MIF expression, high CD74 expression and low MIF expression, and low CD74 expression and high MIF expression. The log‐rank test was repeated to compare the two groups' survival differences pairwise. The top 30% of samples with the highest CD74 expression were defined as the high‐expression group, while the bottom 30% were defined as the low‐expression group. The differential analysis was performed using the “limma” R package to obtain the log2 fold change (log2FC) for each gene. All genes were ranked according to their log2FC, and GSEA enrichment analysis of KEGG gene sets was conducted using the “clusterProfiler” R package. The CancerSEA database compiles 14 different functional states of tumour cells. The *Z*‐score algorithm integrates the expression of characteristic genes to reflect the activity of a given pathway. Using the *Z*‐score parameter in the “GSVA” R package, we computed the combined Z‐scores for the 14 functional state gene sets. We further standardised these scores using the “scale” function to define gene set scores and calculated the Pearson correlation between CD74 and each gene set score.

### 2.6 Establishing the CAF‐Related Signature

A univariate Cox regression analysis was conducted on CAF markers using the “survival” R package to screen out prognosis‐related CAF genes (*p* < 0.05). In the training set, prognosis‐related CAF genes were analysed using Lasso‐Cox regression analysis. Using the “glmnet” R package, the multicollinearity was eliminated. The “glmnet” function was used to perform Cox penalty regression analysis. The “cv.glmnet” function was applied to perform the 10‐fold cross‐validation [29, 30]. In the meta dataset, the minimum partial likelihood deviation estimated from the 10‐fold cross‐validation was used to determine the penalty parameter. Under the specified lambda, the coefficient β (Coef) value for each gene was extracted. Finally, a prognostic risk score model of β (Coef) values multiplied by expression levels of the CAF‐related gene (CAFRG) was established.

### 2.7 Estimating the Accuracy of the CAF‐Related Signature

The risk scores of patients in the GSE21257 dataset were all calculated, on which the univariate Cox regression analysis was conducted. The median of risk scores in the training was set as the cut‐off value, all osteosarcoma patients were divided into the high‐ or low‐risk group. Then, the Receiver operating characteristic (ROC) curve was generated by the R package “timeROC” which was used to calculate the area under the curve (AUC). The Kaplan–Meier (KM) survival curve of the risk scores was plotted via the “survminer” R package. The calibration curve was generated using the “RMS” R package. They were designed to observe whether the CAF‐related prognosis signature could actually and repeatably predict the prognosis of osteosarcoma patients. To enhance the persuasiveness and reliability of our results, three external validation datasets were incorporated. First, the *Z*‐score was used to standardise all datasets to ensure a mean of 0 and a standard deviation of 1, eliminating scale differences between variables for suitable statistical analysis. Next, we applied the exponential function to transform z‐scores into positive values, aligning the data's distribution properties with the subsequent risk assessment model. With this preprocessed data, we calculated risk scores using coefficients from the LASSO‐Cox results. For a more reliable prognostic model, we integrated clinical variables into a multivariate Cox model and constructed a nomogram, evaluating its robustness with the “timeROC” R package and its appropriateness with the “ggDCA” R package. We sourced clinical data from the UCSC Xena database for Target‐OS, merged it with risk scores, and categorised patients as adults or children based on age (cutoff of 14 years). Finally, we conducted Wilcoxon Rank Sum Tests to assess statistical significance.

### 2.8 Excavating Potential Pathway Mechanisms in Osteosarcoma

Using the “clusterProfiler” R package [31, 32], the Gene Set Enrichment Analysis (GSEA) was performed on the hallmark, C2 (cp.KEGG, cp.reactome, cp.wikipathways), C5 (go.bp, go.cc, go.mf, hpo), and CancersFunctionalStates gene sets from the MSigDB to compare the pathway activation differences between the high‐ and low‐risk group. By the “WGCNA” R package, a weighted gene co‐expression network with approximately scale‐free characteristics was constructed [33]. Using the topological overlap matrix (TOM), network modules were then generated [34]. Using the dynamic hybrid cutting method (bottom‐up algorithm), co‐expressed gene modules were identified and similar ones were combined [35]. Gene modules significantly correlated with higher risks were identified. Then, the Kyoto Encyclopedia of Genes and Genomes (KEGG) analysis was performed on all genes in the modules [36, 37].

### 2.9 Describing the Immune Landscape of the TME in Osteosarcoma

To improve the precision, eight different software (xCell [38], TIMER [39, 40], QUANTISEQ [41], MCPcounter [42], IPS, ESTIMATE [43], EPIC [44], CIBERSORT [45]) were applied to depict the immune infiltration landscape in osteosarcoma. Differences of contents of various immune cells between the high‐ and low‐risk group were compared. The Spearman correlation coefficients between the signature (risk score) and contents of immune cells were calculated and displayed.

### 2.10 Analysing Drug Resistance and Predicting Immunotherapy Responses

Eight immunotherapy response scoring hallmarks (IFNγ [46], Tertiary lymphoid structure (TLS) [47], Tcell_inflamed [46], Roh_IS [48], Davoli_IS [49], Cytolytic activity (CYT) [50], chemokines [51], Ayers_expIS [46]) were enrolled. Differential analyses were conducted to compare their values between the high‐ and low‐risk groups. From the Genomics of Drug Sensitivity in Cancer (GDSC) database [52], the half maximal inhibitory concentration (IC50) values of 265 micro‐molecules in 860 cell lineages and corresponding gene mRNA expression were collected. The correlation between mRNA expression levels of certain genes and IC50 values of diverse drugs was analysed by the Pearson correlation analysis.

### 2.11 Statistical Analysis

The Wilcoxon rank sum test was used to check the difference between the two variables. Cox and KM survival analyses were performed using the “Survival” R package. KM survival analysis was tested by log‐rank test. All statistical tests were two‐tailed. The *p*‐value < 0.05 would be considered statistically significant. When *p* < 0.001, the statistical significance would be more significant. Hazard ratios (HRs) and 95% confidence intervals (CIs) described relative risks. R software (V.4.2.2) (Institute for Statistics and Mathematics, Vienna, Austria) was applied.

## 3 Results

### 3.1 Screening out 96 CAF‐Related Genes and Identifying 22 Clusters Annotated to 14 Cell Types

**FIGURE 1:**
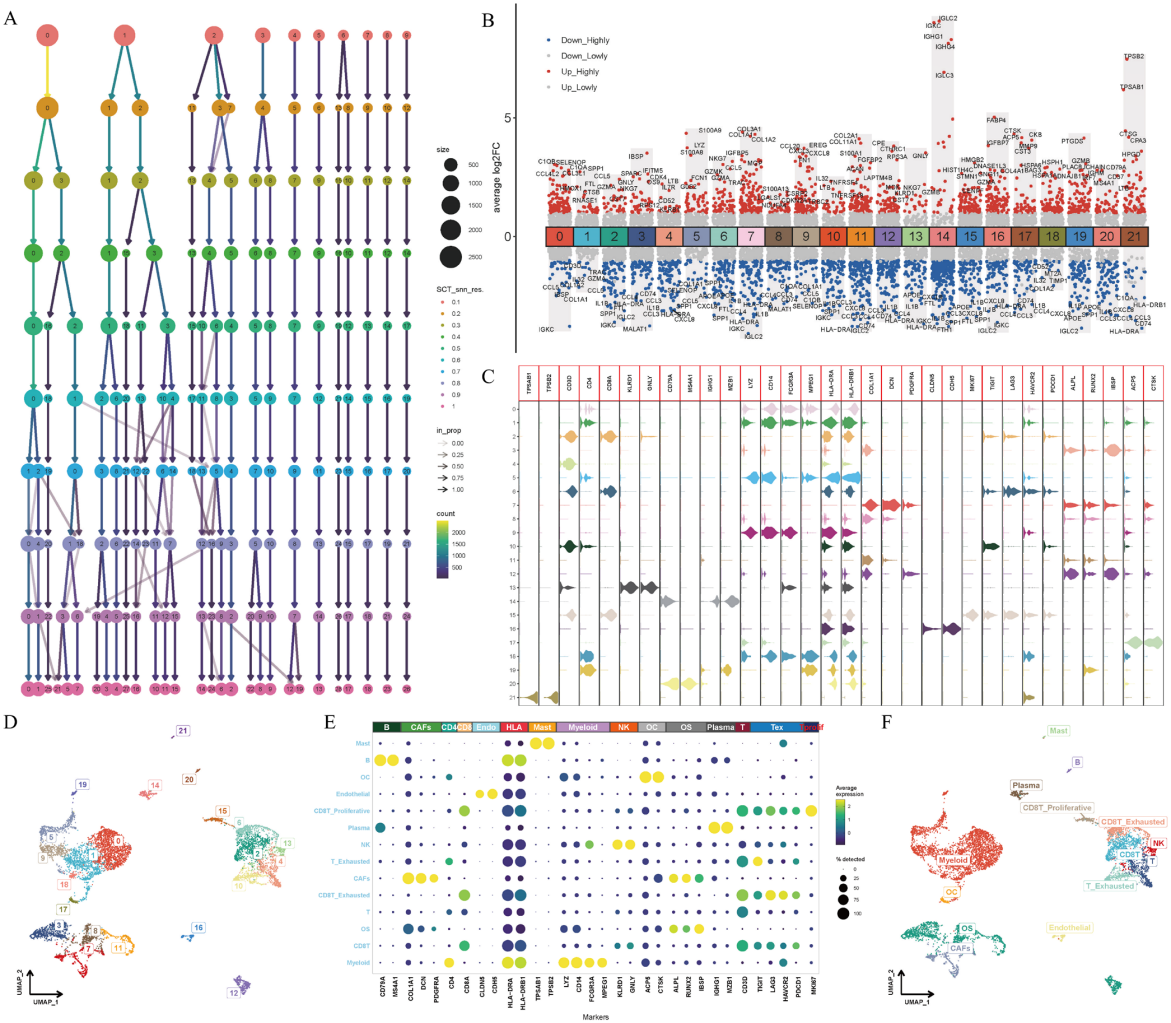
Profiles of cells in the tumour microenvironment of osteosarcoma at the single‐cell level. A clustering tree described the optimal resolution as 0.6 (A). Differentially expressed genes in 22 clusters (B). Lineage‐specific gene expression visualisations in 22 clusters (C). Umap showing the spatial distributions of 22 clusters (D). Marker gene expressions in different clusters (E). Umap reflecting spatial distributions of different cell types in 22 clusters (F).

Based on the GSE162454 dataset, a total of 96 CAF‐related genes were selected. From Figure 1A, the cluster tree displayed correlations between different clusters under different resolutions. 0.6 was the best resolution. 46,544 cells were grouped into 22 clusters. Figure 1B showed the differentially expressed genes in each cluster. Figure 1C showed genes that could reflect some of the cell lineage features in different clusters. In Figure 1D, the spatial distribution of 22 clusters in various cells was visualised in uniform manifold approximation and projection (UMAP). Based on the specific lineage genes, manual annotations were completed. From Figure 1E, the distributions of different markers were shown. In Figure 1F, results of manual annotations were displayed. The 0, 1, 5, 9, 18, and 19 clusters were annotated as myeloid cells. The 2 cluster was annotated as a CD8T cell. The 3, 8, 11, and 12 clusters were annotated as osteosarcoma cells. The 4 cluster was annotated as a T cell. The 6 cluster was annotated as an exhausted CD8 T cell. The 7 cluster was annotated as CAFs. The 10 cluster was annotated as an exhausted T cell. The 13 cluster was annotated as a NK cell. The 14 cluster was annotated as a plasma cell. The 15 cluster was annotated as a proliferative CD8 T cell. The 16 cluster was annotated as an endothelial cell. The 17 cluster was annotated as osteoclast. The 20 cluster was annotated as a B cell. The 21 cluster was annotated as a mast cell.

### 3.2 The Landscape of Cell Subpopulations and Potential Mechanisms Analysed at the Single‐Cell Level

**FIGURE 2:**
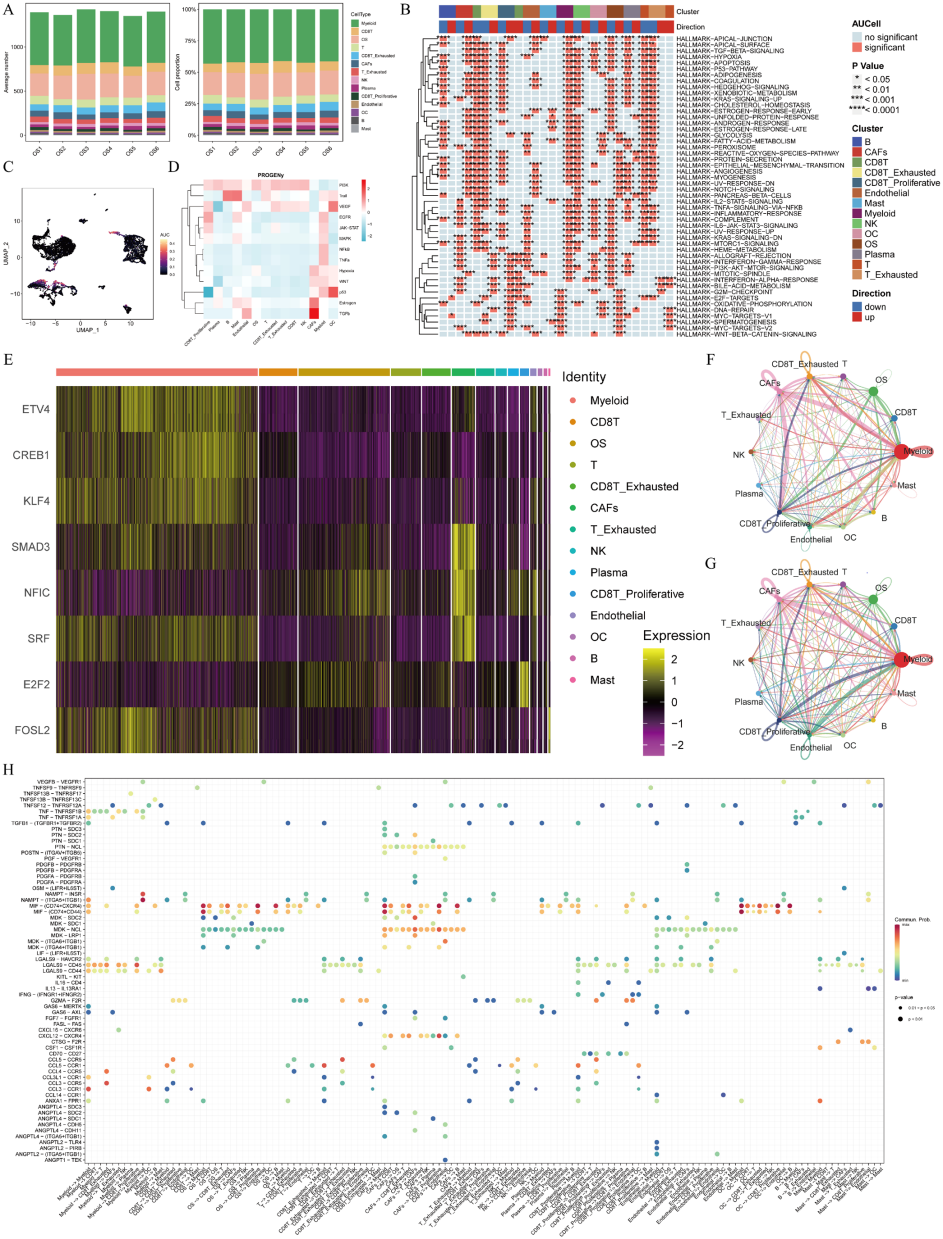
Profiles of potential mechanism analyses at the single‐cell level. Bar charts showing the average number and proportion of 14 various cell types in six OS patients from the single‐cell dataset (A). The heatmap showing the scores of different hallmark gene sets in different cells (B). Umap exhibiting the spatial distributions of a defined‐proliferous gene set in different cell populations (C). The heatmap comparing the PROGENy scores of cells in different signalling pathways (D). The eight transcription factors' activity differences in various cells (E). Cell–cell interaction networks between multiple main cell types based on interaction numbers and strength (F, G). An overall view of ligand‐receptor interactions in different cells (H).

From the GEO database, a single‐cell dataset containing six osteosarcoma patients and 46,544 cells to dissect the TME profiles. Figure 2A showed the average number and proportion of the 14 different cells (myeloid cells, CD8T cells, osteosarcoma cells, T cells, exhausted CD8 T cells, CAFs, exhausted T cells, NK cells, plasma cells, proliferative CD8 T cells, endothelial cell, osteoclasts, B cells, mast cell) in six osteosarcoma patients (named as OS1, OS2, OS3, OS4, OS5, and OS6). Figure 2B showed the score of the hallmark gene set in the 14 cells singly. Figure 2C reflected the UMAP of the distribution of the proliferative gene set in the 14 cells. The heatmap displayed PROGENy scores of the 14 cells in 13 various types of signalling pathways (Figure 2D). The PI3K signalling pathway was activated in most of the 14 cells. P53 and VEGF signalling pathways were activated in osteoclast (OC) cells. TGFb and Oestrogen pathways were activated in CAFs. The another heatmap showed the transcription factor activity of eight TFs in the 14 cells (Figure 2E), showing the cell‐specificity of eight TFs. The activity of SMAD3, NFIC, and SRF in CAFs was relatively higher compared to other cells. Figure 2F,G counted overall conception of cell–cell communication networks. The upper circle diagram showed the numbers of interactions between different cell populations. The nether circle diagram showed the strength (probability) of cell–cell interactions. The bubble diagram visualised the overall view of multiple ligand‐receptor interactions between different cells (Figure 2H). The MIF signalling pathway may contribute much to cell–cell interactions.

### 3.3 Further Interpretation of the Complicated Cell–Cell Interaction Networks From Various Angles

**FIGURE 3:**
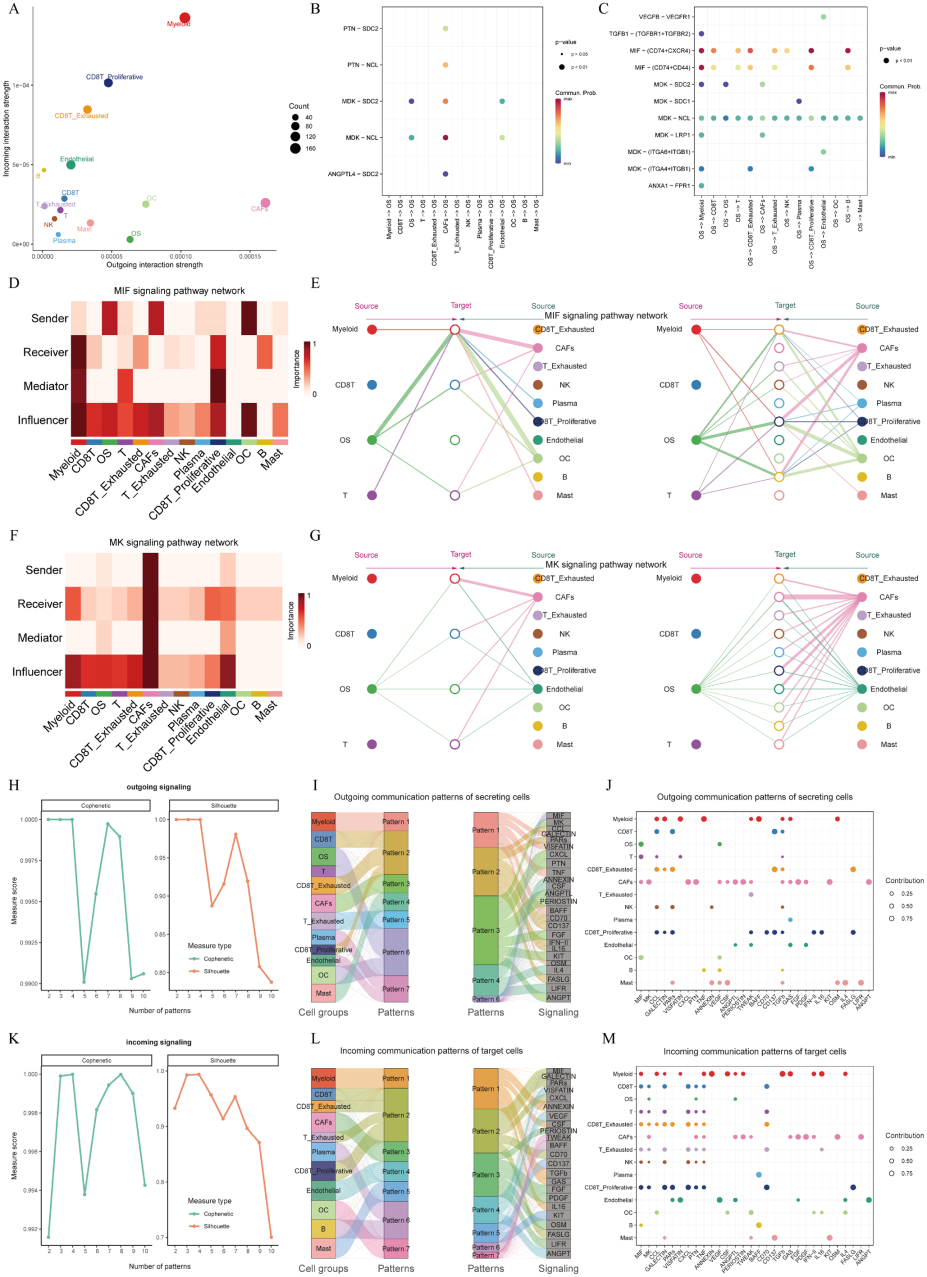
CAFs play as a key regulator in OS progression. Scatter plots indicating CAFs as the strongest factor in both outgoing and incoming interactions (A). Bubble diagrams indicating CAFs as important factor in five signalling pathways (B). The MIF and MK signalling pathways were identified as relatively important pathways (C). Heatmaps showing the roles of main types of cells in the MIF and MK signalling pathways (D, F). The hierarchy plot of MIF signalling pathway in OS (E). The MK signalling pathway network in OS (G). NMF curves showing the optimal cluster numbers in outgoing and incoming interactions (H, K). River plots (I, L) and scatter plots (J, M) showing the interaction patterns of different cells in various patterns.

First of all, the scatter diagram (Figure 3A) showed that CAFs had the strongest interaction strength in both the incoming and outgoing interactions among all 14 clusters. To explore the potential factor that may stimulate OS cells, the bubble gram showed that the CAFs‐OS pair had the highest regulation probability in all five signalling pathways (Figure 3B). Figure 3C showed the regulating differences of all ligand‐receptor pairs in various signalling pathways. The MIF and MDK (MK) signalling pathways were the relatively important ones. Next, in Figure 3D,F, the heatmaps showed the importance of the 14 cell subgroups respectively as sender, receiver, mediator, or influencer in MIF and MK signalling pathway networks. The upper one could tell myeloid cells were the most important cell type in all four roles. And CAFs were very important as senders and influencers. The bottom map showed that CAFs had the predominance as all four roles in MK signalling pathway network. Again, the importance of CAFs was emphasised. In hierarchy plot (Figure 3E,G), the ligand‐receptor pairs that contributed most to the MIF and MK signalling pathways were shown. It could be observed that CAFs acted as a vital role as a sender or receiver in both signalling pathways.

In addition, we utilised a function “selectK” to infer the number of outgoing and incoming signalling patterns based on the two metrics Cophenetic and Silhouette that have been implemented in the “NMF” R package. From Figure 3H,K, the point that Cophenetic and Silhouette values both acted well was selected (*K* = 7). Two river plots (Figure 3I,L) showed outgoing communication patterns of secreting cells and incoming communication patterns of target cells. Two scatter plots displayed outgoing communication patterns of secreting cells and incoming communication patterns of target cells in a different way (Figure 3J,M).

### 3.4 CAFs May Contribute to the Oncogenic Characteristics of Sarcomas Through the MIF‐CD74 Signalling Axis

The results demonstrated that MIF was dysregulated in the majority of tumours (Figure S1A). Similarly, MIF was highly expressed in sarcomas like in other tumours (Figure S1B). MIF interacted with CD74 (Figure S1C). In the TCGA database, there was no significant difference in survival between the high MIF expression and low CAFs infiltration group and the low MIF expression and low CAFs infiltration group. However, the presence of both high CAFs infiltration and high MIF expression was associated with poorer survival than high CAFs infiltration alone, indicating a biological connection between them (Figure S1D,E). In the GSE21050 dataset, the group with both high CD74 and MIF expressions had the worst prognosis, suggesting the potential of a pro‐oncogenic signalling axis involving CD74 and MIF (Figure S1F,G). GSEA results indicated that numerous pathways related to inflammation, metabolism, and environmental information processing were enriched in the high CD74 expression group (Figure S1H). Besides, CD74 was primarily associated with pro‐inflammatory and metastatic features (Figure S1I,J). In Figure S2, the oncogenic characteristics of CD74 were evaluated, and all results were highly consistent, suggesting CAFs may promote sarcoma oncogenesis via the MIF‐CD74 signalling axis.

### 3.5 The 11‐CAF Signature Possessed Satisfying Predictive Power of the Prognosis in Osteosarcoma Patients

**FIGURE 4:**
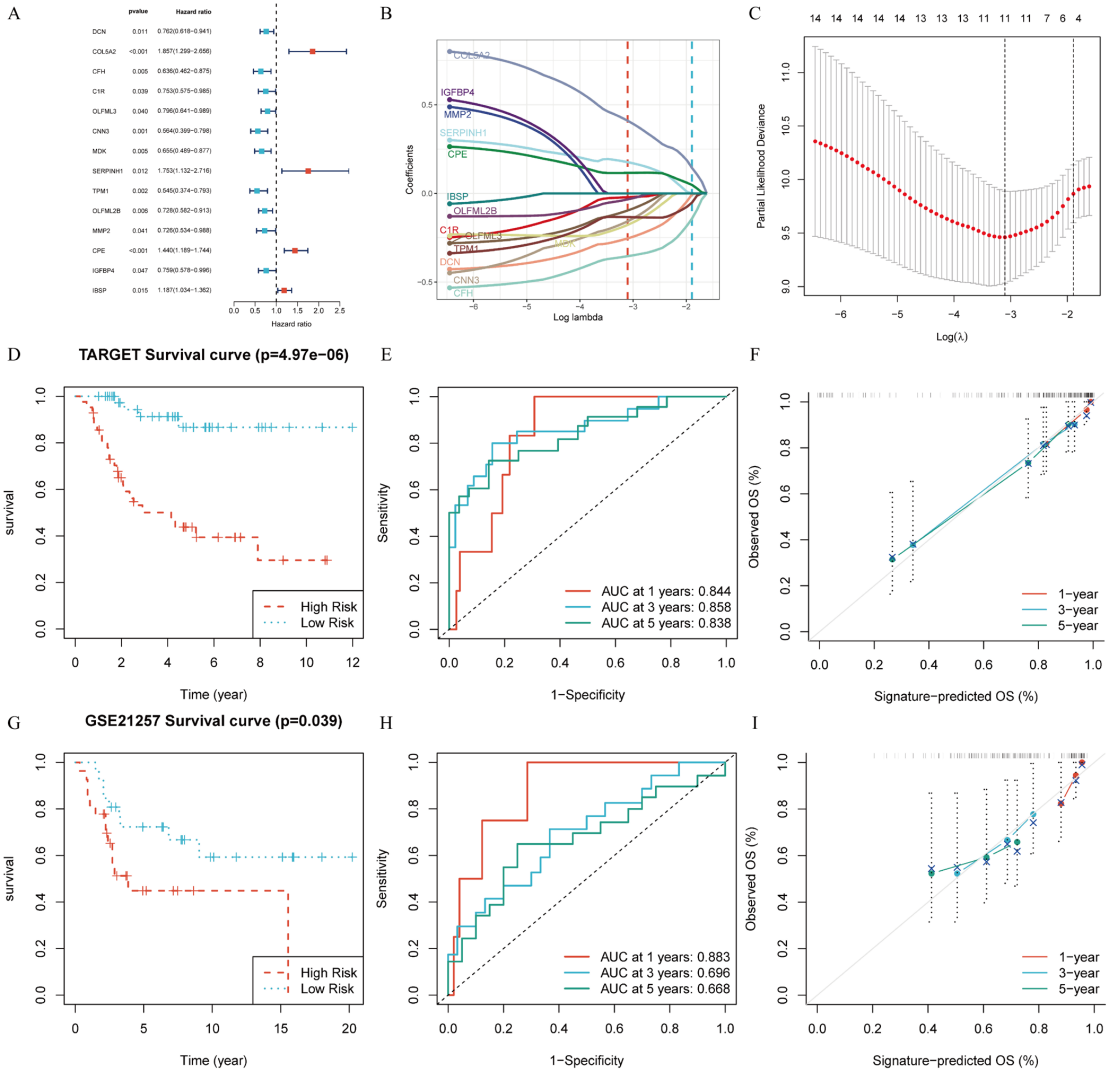
Construction and evaluation of a CAF‐related prognostic signature. Univariate Cox regression analysis revealed 14 prognostic CAF genes in OS (A). Partial likelihood deviance of variables revealed by the Lasso regression model (B). Coefficient values of each signature gene (C). KM, ROC, and calibration curves indicating the accuracy and robustness of the signature in both training and validation datasets (D–I).

Using the univariate Cox analysis, 14 prognosis‐related CAF‐related genes (CAFRGs) were identified (Figure 4A). Conducting the Lasso‐Cox analysis, 11 key CAFRGs were chosen to constitute a novel prognostic signature, and their coefficient values were shown (Figure 4B,C). To estimate the accuracy and reliability of the signature, KM curves, ROC curves, and calibration curves were plotted. In the training set TARGET, the survival of the low‐risk group was better than that of the high‐risk group (Figure 4D). In Figure 4E, the area under curve (AUCs) values of 1‐, 3‐, and 5‐year survival predicted by the signature were 0.844, 0.858, and 0.838, respectively, showing the signature was stable and dependable. The calibration curve showed that the forecasted survival was consistent with the observed value, which affirmed the signature's predictive power (Figure 4F). The same validations were conducted on the validation dataset GSE21257 (Figure 4G–I). Similar results were observed. Therefore, the new prognosis signature was verified as a robust and credible tool for predicting the clinical outcomes of osteosarcoma patients. Three external validation datasets were enrolled and the results consistently showed that the low‐risk group has better survival outcomes. Five independent datasets were included to validate the robustness of the signature (Figure S3). As shown in Figure S4A, three variables—gender, age, and the presence of metastasis were applied in the training cohort, and the multivariate Cox analysis was conducted to construct a nomogram. To assess the nomogram's robustness in predicting 1‐, 3‐, and 5‐year patient survival rates, we utilised three parameters from the “timeROC” R package (Figure S4B–D). The results showed enhanced predictive performance of the model after incorporating clinical characteristics, with consistent evaluation parameters. Furthermore, we assessed whether the model could benefit patients using the “ggDCA” R package (Figure S4E). In terms of short‐term survival prediction, the nomogram could offer greater benefits to patients compared to other models. However, when it comes to long‐term survival prediction, the original model proves to be the most effective approach. Results showed no significant differences in risk scores between adults and children or between genders, but a significant difference was observed between patients with and without metastasis (Figure S5A–C). External validation confirmed these findings (Figure S5D–F). Consistent with prior research, CAFs play a crucial role in the tumour microenvironment, promoting tumour progression. The significant risk score difference between the metastasis and non‐metastasis groups in our study supports the accuracy of the CAF prognostic model.

### 3.6 Differential Pathways in the High‐ and Low‐Risk Group

**FIGURE 5:**
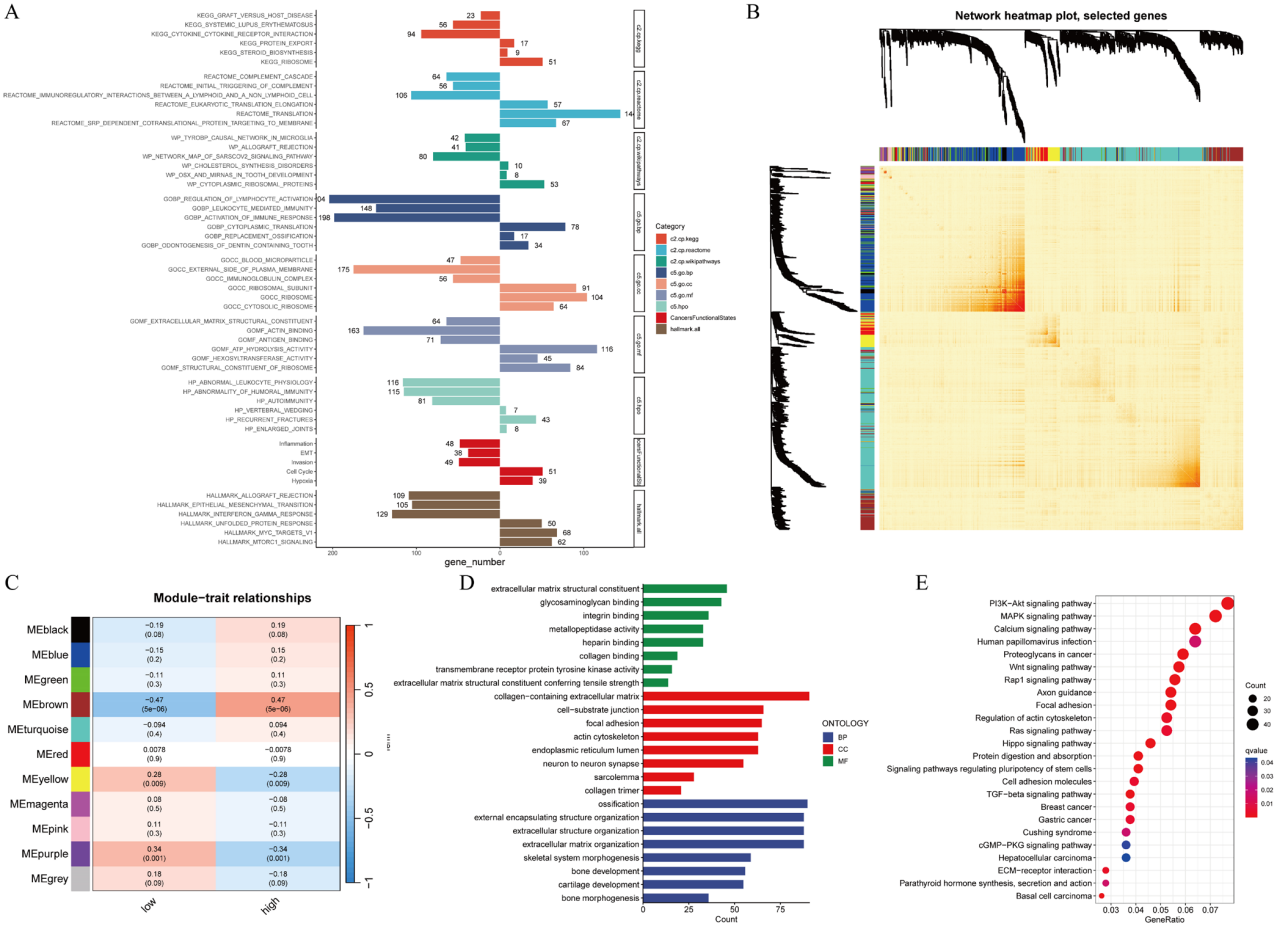
Potential risk mechanism exploration. Bar charts showing GSEA results (A). WGCNA methods providing a dendrogram with coloured bands indicated different modules (B). The brown module was significantly related to higher risk scores (C). GO and KEGG results of the genes from the brown module (D, E).

Bar charts showed GSEA results (Figure 5A). Genes in the high‐ and low‐risk were enriched in differential pathways. For example, KEGG_CYTOKINE_CYTOKINE_RECEPTOR INTERACTION, REACTOME_IMMUNOREGULATORY_INTERACTIONS_BETWEEN_A_LYMPHOID_AND_A_NON_LYMPHOID_CELL, GOBP_REGULATION_OF_LYMPHOCYTE_ACTIVATION, GOBP_LEUKOCYTE_MEDIATED_IMMUNITY, GOBP_ACCTIVATION_OF_IMMUNE_RESPONSE, GOCC_IMMUNOGLOBULIN_COMPLEX, GOMF_ANTIGEN_BINDING, HP_AUTOIMMUNITY, inflammation, and HALLMARK_INTERFERON_GAMMA_RESPONSE and some other immune‐related gene sets were observed more in the low‐risk group, which helped explain why the low‐risk group had better clinical outcomes. The heatmap visualised the gene network and displayed the TOM for all genes (Figure 5B). Totally, 11 gene modules were identified because they had close relations with risk grouping (Figure 5C). The brown module was considered to be the most significant one to the high‐risk group. Next, Gene Ontology (GO) and KEGG analysis were conducted to verify the GSEA results. Figure 5D,E showed the pathways that genes in the brown modules were mainly enriched.

### 3.7 More Immune Infiltration Was Observed in the Low‐Risk Group

**FIGURE 6:**
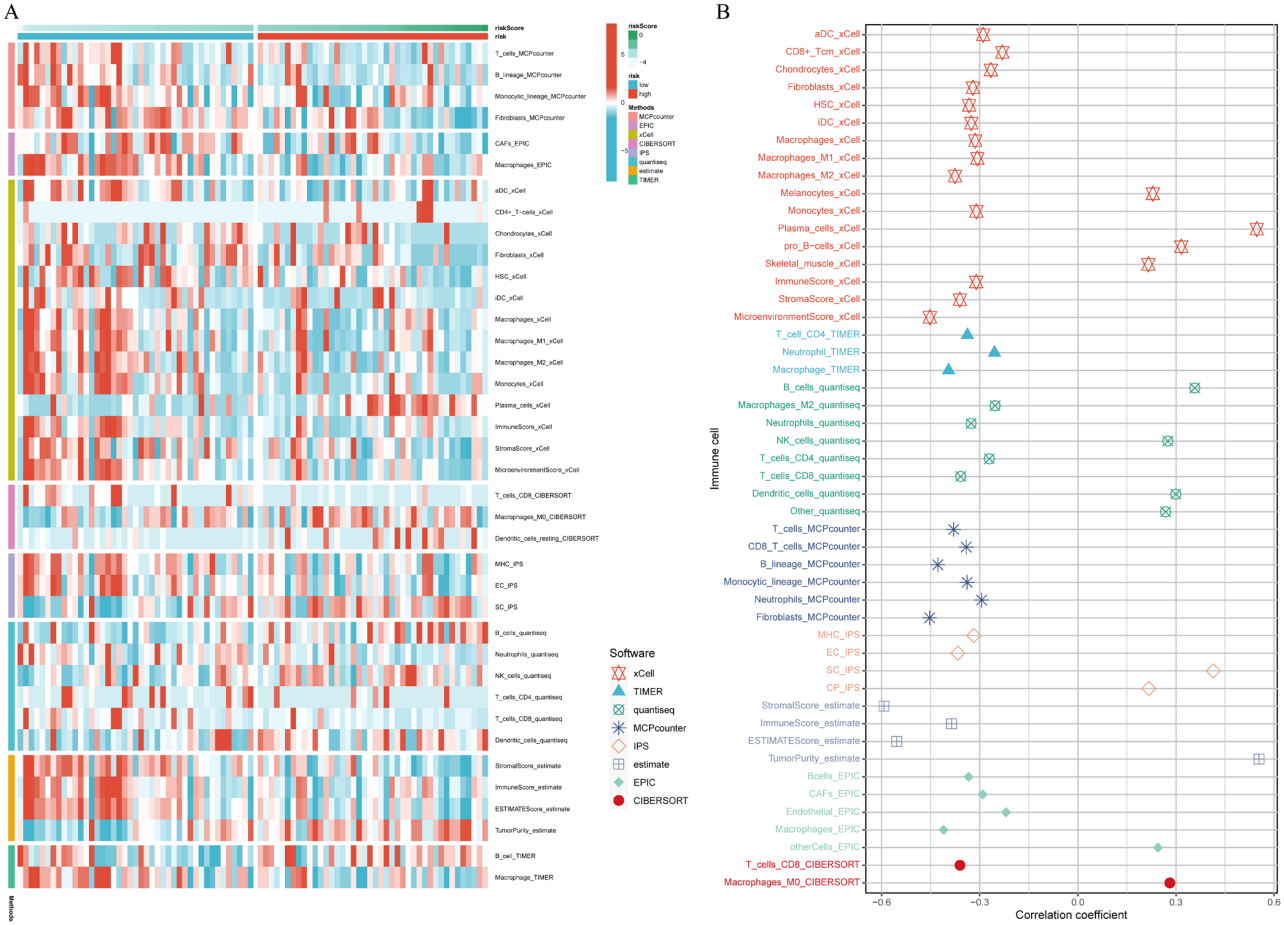
Immune infiltration landscape. Differential immune infiltrating cell numbers between the high‐ and low‐risk group (A). The correlations between immune cells and risk scores (B).

To describe the immune infiltration landscape between the high‐ and low‐risk groups, eight various software were applied. Contents of different immune cells were compared between the high‐ and low‐risk groups using the Wilcoxon rank sum test. Figure 6A showed that the low‐risk group was infiltrated with more immune cells. For example, the MCPcounter approach showed that T cells and B cells were expressed more in the low‐risk group. EPIC showed that CAFs were expressed more in the low‐risk group. Then, Spearman correlation analysis was conducted and provided similar results. Figure 6B showed that risk scores were significantly negatively related to the vast majority of immune cells. The concentration of T cells was negatively correlated with risk scores according to the analytic results of xCell, TIMER, quantiseq, MCPcounter, and CIBERSORT. The concentration of B cells was negatively correlated with risk scores according to the analytic results of xCell, quantiseq, MCPcounter, and EPIC. The concentration of CAFs was negatively correlated with risk scores according to the analytic result of EPIC.

### 3.8 The Low‐Risk Group Was More Likely to Respond to Immunotherapy and Chemotherapy

**FIGURE 7:**
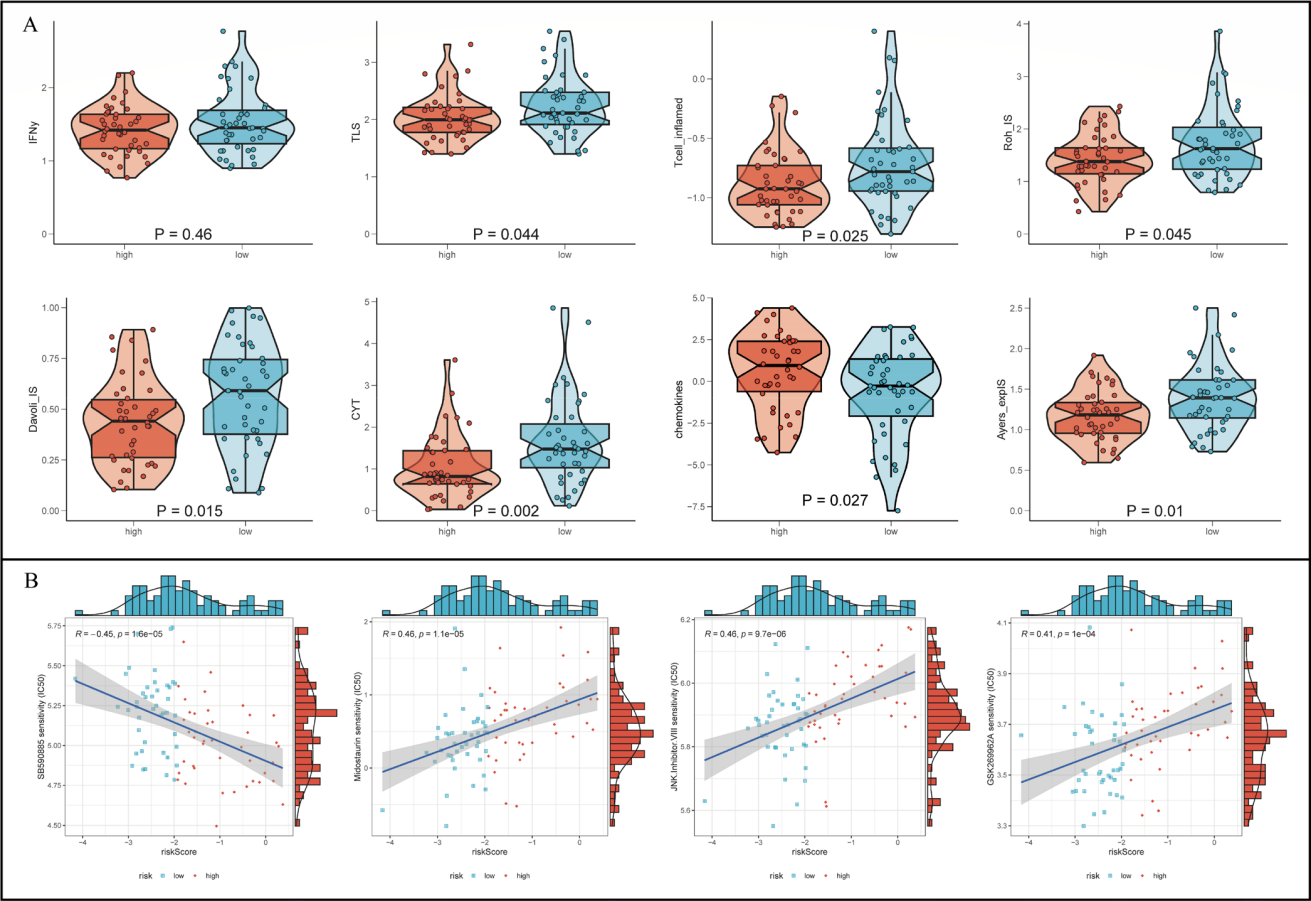
Application of the signature in guiding treatments. Differential expression levels of eight traditional biomarkers for immunotherapy in OS between the high‐ and low‐risk group (A). Correlations between IC50 values of four kinds of chemotherapy drugs and risk scores (B).

To verify differential expression levels of some known immunotherapy biomarkers between the high‐ and low‐risk groups, a differential analysis was conducted. Figure 7A showed that TLS, Tcell_inflamed, Davoli_IS, Cytolytic activity (CYT), Ayers_expIS were expressed more in the low‐risk group (*p* < 0.05). On the contrary, chemokines were expressed more in the high‐risk group (*p* = 0.027). Moreover, IC50 values of midostaurin, JNK.Inhibitor.VII, and GSK269962A were positively correlated with risk scores. And the SB590885 IC50 value was negatively related to risk scores (Figure 7B). All results demonstrated that the low‐risk group patients were more suitable to be treated with immunotherapy and chemotherapy.

## 4 Discussion

Osteosarcoma (OS) is a tumour of mesenchymal origin [53], mainly affecting children and young adults [54]. With a high propensity for invasion and metastasis, OS is known as a highly malignant disease. Therefore, finding effective treatments for OS is of great significance in improving patients' quality of life and long‐term prognosis.

Along with the development of modern medicine, the management of OS has been diversified. Multi‐therapies have been introduced. For example, the basis is the timely use of surgical resection and neo‐adjuvant and adjuvant chemotherapy, but radiotherapy is not efficient due to the high radioresistance in osteosarcomas [2]. Even though the good combination of surgery and chemotherapy has greatly improved the clinical outcomes of OS patients to some extent, it is inadequate and unsatisfactory for metastatic or recurrent OS patients. Immunotherapy has been proven to be a promising way to treat cancers for it could enhance the prognosis of cancer patients. Suffering from a lack of robust biomarkers to screen out individuals who are suitable to accept immunotherapy and to make a personalised medication regimen, the effect of immunotherapy has been highly restricted. Hence, the aim of this study was to build a novel prognostic signature as to provide a potential biomarker for the treatment protocol making and prognosis predicting.

First of all, we processed the raw data and classified all cells into 22 clusters using the scRNA‐seq technology to explore the immune heterogeneity of OS cells and dissect the precise cellular compositions in the TME of OS. The 22 clusters were annotated manually to 14 different kinds of cell subpopulations. The indispensable role of CAFs has been highlighted then. The crosstalk between tumour cells and stromal cells in the TME has been considered a vital interaction that contributes to tumour initiation and progression. CAFs, consisting of more than 50% of all stromal cells, are a key component in the TME of cancers, providing a favourable environment for tumour cell growth [55]. The abundance of CAFs has been confirmed to be related to prognosis among different human cancers, and consequently, CAFs have also been regarded as a therapy target that can modulate the efficacy of therapies and influence the clinical outcomes of patients [56]. With the aid of the “cellchat” R package, the signalling ligand–receptor interactions were analysed and visualised. We revealed the intercellular link and CAFs were validated as having a multidimensional role in both the outgoing and incoming signalling pathways of OS proliferation.

Using the Univariate Cox regression analysis, there were 14 CAF‐related genes (CAFRGs) (COL5A2, IGFBP4, MMP2, SERPINH1, CPE, IBSP, OLFML2B, C1R, OLFML3, TPM1, MDK, DCN, CNN3, CFH) related to the prognosis of OS obtained. After that, the Lasso‐Cox regression analysis was conducted to determine 11 hub CAFRGs (COL5A2, SERPINH1, CPE, OLFML2B, C1R, OLFML3, TPM1, MDK, DCN, CNN3, CFH). Some of these genes have already been explored to have varying degrees of impact on the biological processes of OS cells. For example, COL5A2 has been reported to inhibit the TGF‐β and Wnt/β‐Catenin Signalling Pathways to prohibit the invasion and metastasis of OS [57]. COL5A2 may be associated with the aggressiveness and metastasis of osteosarcoma, affecting immune cells in the tumour microenvironment [58]. Besides, the expression of TPM1 may be related to the aggressiveness of the tumour and the regulation of the immune microenvironment, which may affect the immune surveillance of human oral squamous cell carcinoma [59]. Jiang et al. [60] have demonstrated that MiR‐107 overexpression promoted OS cell viability and migration/invasion via downregulation of TPM1. In addition, DCN coating on titanium surface simultaneously inhibited the oncogenic potential of osteosarcoma cells but enhanced cell growth of pre‐osteoblasts [61]. CPE is a key regulator of growth and metastasis in multiple cancer types [62], and in OS CPE promotes migration, invasiveness, and epithelial‐mesenchymal transition of OS cells via the Wnt‐β‐catenin pathway [63]. Hence, silencing CPE was testified to be favourable for OS growth [64]. SERPINH1 has been validated to function as a tumorigenic and immunogenic gene [65]. Xia et al. [66] demonstrated that SERPINH1 promoted the proliferation, migration and invasion of osteosarcoma cells and promoted the growth of osteosarcoma in vivo by activating the PI3K‐Akt signalling pathway. Also, CNN3 has been regarded as a risk factor in OS cells, that is, CNN3 may play an oncogenic role during the progression of osteosarcoma by activating the ERK1/2 and p38 pathways [67]. Next, based on these 11 genes, we used the formula “risk score = (β1*CAFRG1 + β2*CAFRG2 + β3*CAFRG3 + ⋯ + βn*CAFRGn)”. β referred to the coefficient values of CAFRGs [29, 30]. The median value of risk score was seen as a cut‐off to divide all OS patients into either the high‐ or low‐risk group. The TARGET dataset and GSE21257 were used as the training set and validation set. KM survival analysis, ROC curves, and calibration curves were conducted on the two sets. Similar results were obtained to show that, based on the prognosis signature, patients in the low‐risk group would have better clinical outcomes. The accuracy and robustness of the signature were confirmed accordingly. From the GSEA and WGCNA results, we observed that more immune‐related gene sets were enriched in the low‐risk group. Using eight different approaches, we identified that the low‐risk group was infiltrated with more immune cells. Moreover, the differential analysis and correlation analysis showed that the low‐risk group patients were more suitable to accept immunotherapy and chemotherapy, which explained why the low‐risk group could live longer. All results again proved that the signature was reliable.

Nevertheless, several limitations should be acknowledged. First, all data were downloaded from public databases, making sure that the CAF clusters and CAF‐based risk signatures were generated using retrospective data. Also, we lacked treatment and relapse records. Therefore, in the future, our conclusions should be validated experimentally. Secondly, we only investigated the potential prognostic value of the CAF‐related risk signature. Therefore, further studies are needed to explore and define the underlying mechanisms of the signature in the development of OS.

## 5 Conclusion

Taken together, the CAFRGs have been illustrated to have great impacts on OS, and an 11‐gene prognostic signature was proposed to predict the prognosis in patients with OS.

## Author Contributions

**Yukang Que:** conceptualization (lead), data curation (lead), formal analysis (lead), writing – original draft (lead). **Tianming Ding:** formal analysis (supporting), validation (supporting). **Huming Wang:** data curation (supporting), formal analysis (supporting), funding acquisition (supporting). **Shenglin Xu:** investigation (supporting), methodology (supporting), visualization (supporting). **Peng He:** formal analysis (supporting), funding acquisition (supporting), visualization (supporting). **Qiling Shen:** data curation (supporting), project administration (supporting). **Kun Cao:** resources (supporting), validation (supporting). **Yang Luo:** formal analysis (supporting), methodology (supporting), visualization (supporting). **Yong Hu:** funding acquisition (lead), project administration (equal).

## Conflicts of Interest

The authors declare no conflicts of interest.

## Supporting information


**Figure S1.** Verification of the CAFs’ contribution to sarcoma oncogenic characteristics via the MIF‐CD74 signalling axis. Gene expression differences between tumour and normal tissues in cancer cohorts. Box plots show quartiles, with the line indicating the median. Wilcoxon Rank Sum Tests compare expressions between groups (A). The lollipop plot shows MIF expression (nTPM) across different tissues, with points representing gene expression in cancer cell lines (B). Scatter plot of potential interacting proteins centered on MIF, with coloured lines indicating subcellular localization evidence (C). Z‐score scatter plots of samples, coloured by subgroup, comparing MIF and fibroblast scores. *Z*‐scores ≤ 0 indicate low expression/scores, and > 0 indicate high (D–F). Kaplan–Meier survival analysis with log‐rank tests. Significant *p*‐values (< 0.05) are highlighted in grey‐backed tables (E–G). The bar plot summarises pathways significantly enriched in CD74 high/low expression groups, with colour indicating enrichment direction (H). Scatter plots show a correlation between functional status *z*‐scores and gene expression z‐scores, with colour indicating functional status type and R indicating Pearson correlation coefficient (I, J).
**Figure S2.** Validation of scores of 14 tumour status across multiple external sarcoma datasets. Scatter plots show functional status z‐scores vs. gene expression *z*‐scores, coloured by functional status type, with *R* indicating Pearson correlation coefficient. Dataset names are labelled.
**Figure S3.** Further external dataset validation of the prognostic model using Kaplan–Meier survival analysis. Dataset names are in brackets above plots. Red/blue curves represent high/low risk groups. Significant *p*‐values (< 0.05) from Log‐rank tests are noted.
**Figure S4.** Enhancing model robustness with a nomogram. The nomogram visually represents variable contributions to OS prediction, with total points estimating survival probabilities (A), and includes time‐dependent ROC curves at 1, 3, and 5 years to illustrate model performance over time (B–D), as well as decision curve analysis (DCA) showing the net benefit of the nomogram compared to alternatives across threshold probabilities (E).
**Figure S5.** Association between model and clinical variables. Risk score differences across clinical variables in the Target‐OS dataset (A–C). Risk score differences across clinical variables in the GSE21257 dataset (D–F). Box plots show quartiles (Q1 and Q3) with whiskers extending to 1.5× IQR from Q1 and Q3.

## Data Availability

The data that support the findings of this study are available from the corresponding author upon reasonable request.
