# Supplementary material for: Prognostic and Therapeutic Significance of Cancer‐Associated Fibroblasts Genes in Osteosarcoma Based on Bulk and Single‐Cell RNA Sequencing Data
Source: J Cell Mol Med. 2025 Mar 5;29(5):e70424. doi: 10.1111/jcmm.70424 (PMC11882394; doi:10.1111/jcmm.70424)

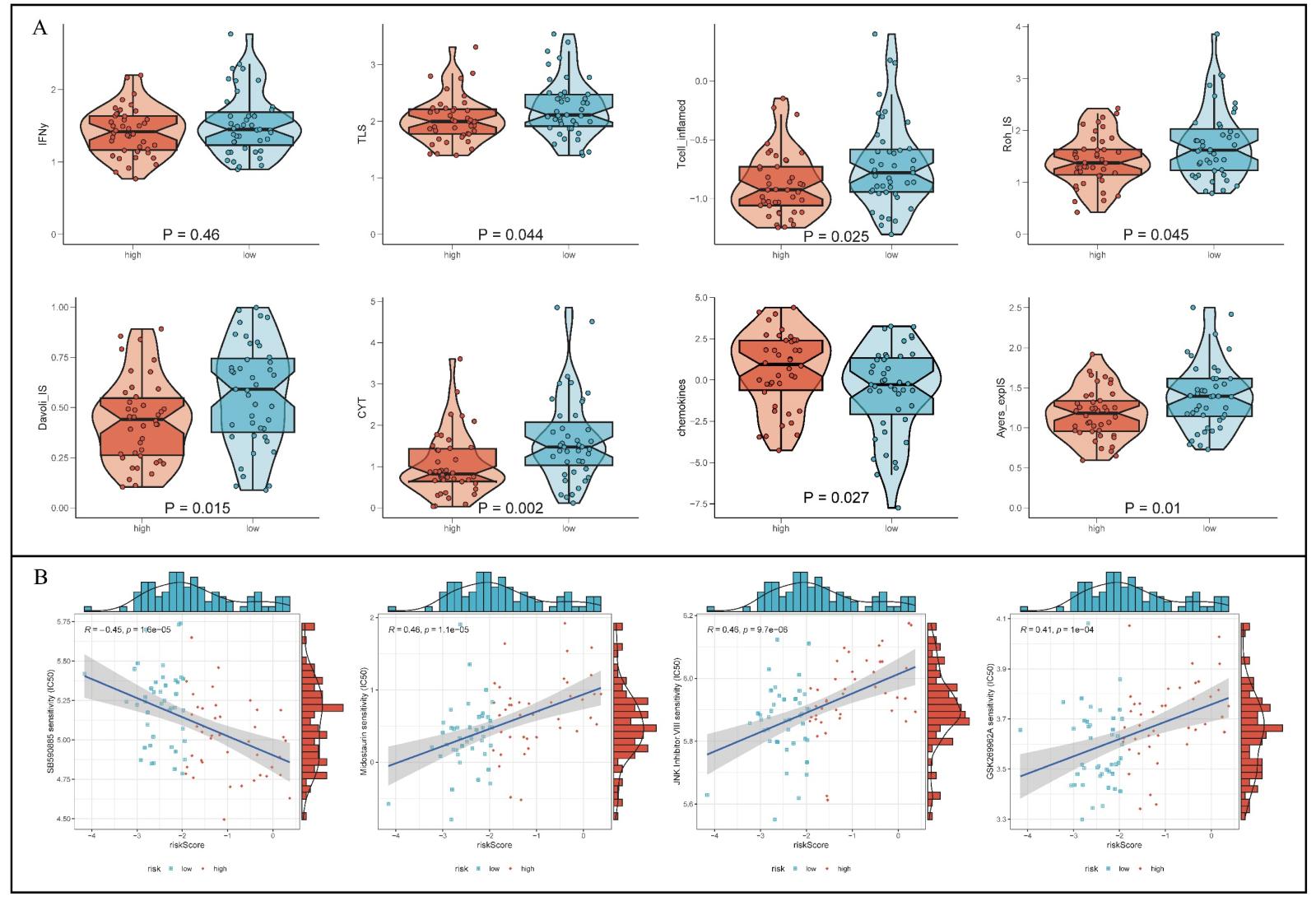


**Supplementary Figure 1**: Verification of the CAFs' contribution to sarcoma oncogenic characteristics via the MIF-CD74 signaling axis. Gene expression differences between tumor and normal tissues in cancer cohorts. Box plots show quartiles, with the line indicating the median. Wilcoxon Rank Sum Tests compare expressions between groups (A). The lollipop plot shows MIF expression (nTPM) across different tissues, with points representing gene expression in cancer cell lines (B). Scatter plot of potential interacting proteins centered on MIF, with colored lines indicating subcellular localization evidence (C). Z-score scatter plots of samples, colored by subgroup, comparing MIF and fibroblast scores. Z-scores ≤ 0 indicate low expression/scores, and>0 indicate high (D-F). Kaplan-Meier survival analysis with Log-rank tests. Significant p-values (<0.05) are highlighted in gray-backed tables (E-G). The bar plot summarizes pathways significantly enriched in CD74 high/low expression groups, with color indicating enrichment direction (H). Scatter plots show a correlation between functional status z-scores and gene expression z-scores, with color indicating functional status type and R indicating Pearson correlation coefficient (I-J).


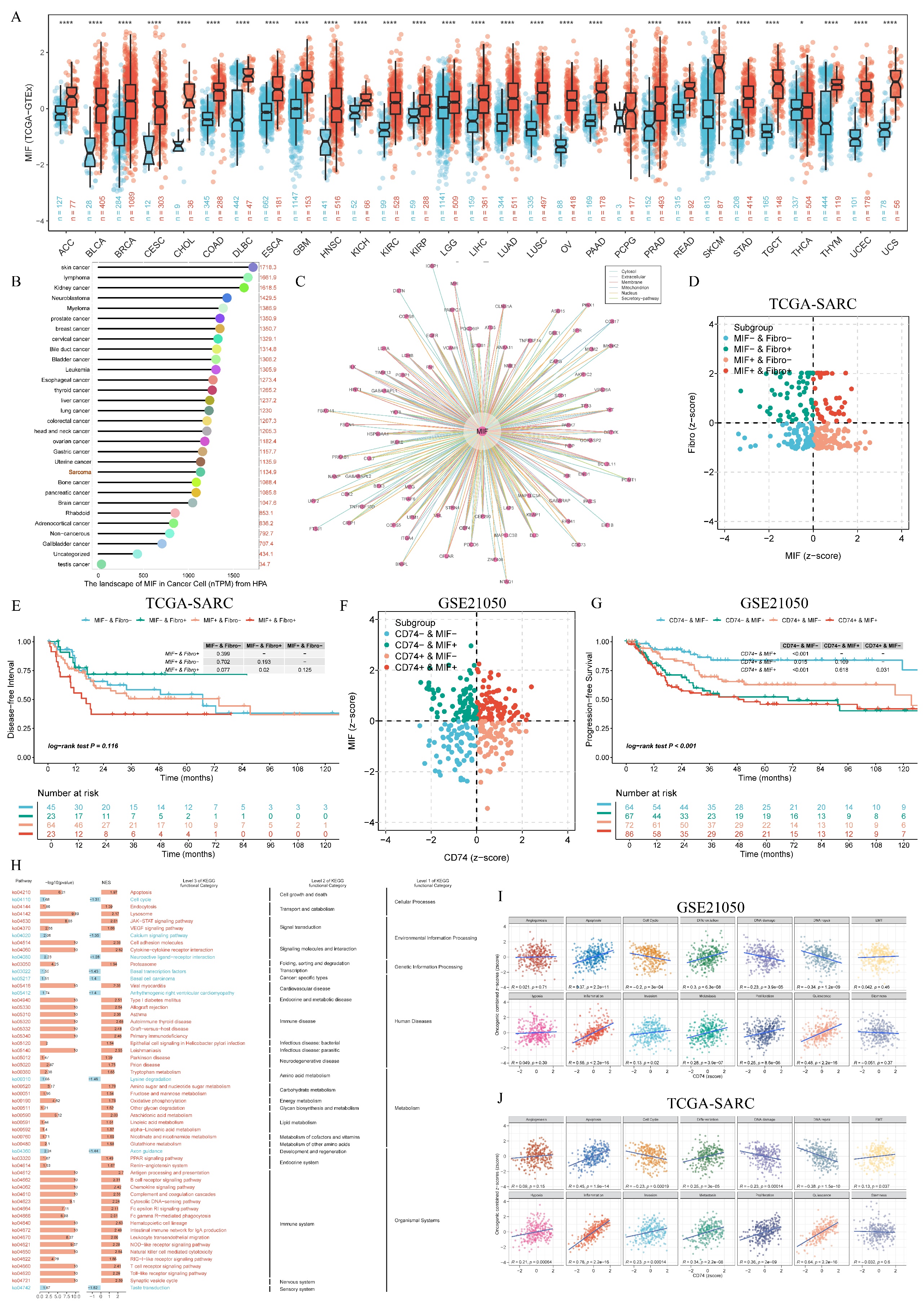


**Supplementary Figure 2**: Validation of scores of 14 tumor status across multiple external sarcoma datasets. Scatter plots show functional status z-scores vs. gene expression z-scores, colored by functional status type, with R indicating Pearson correlation coefficient. Dataset names are labeled.


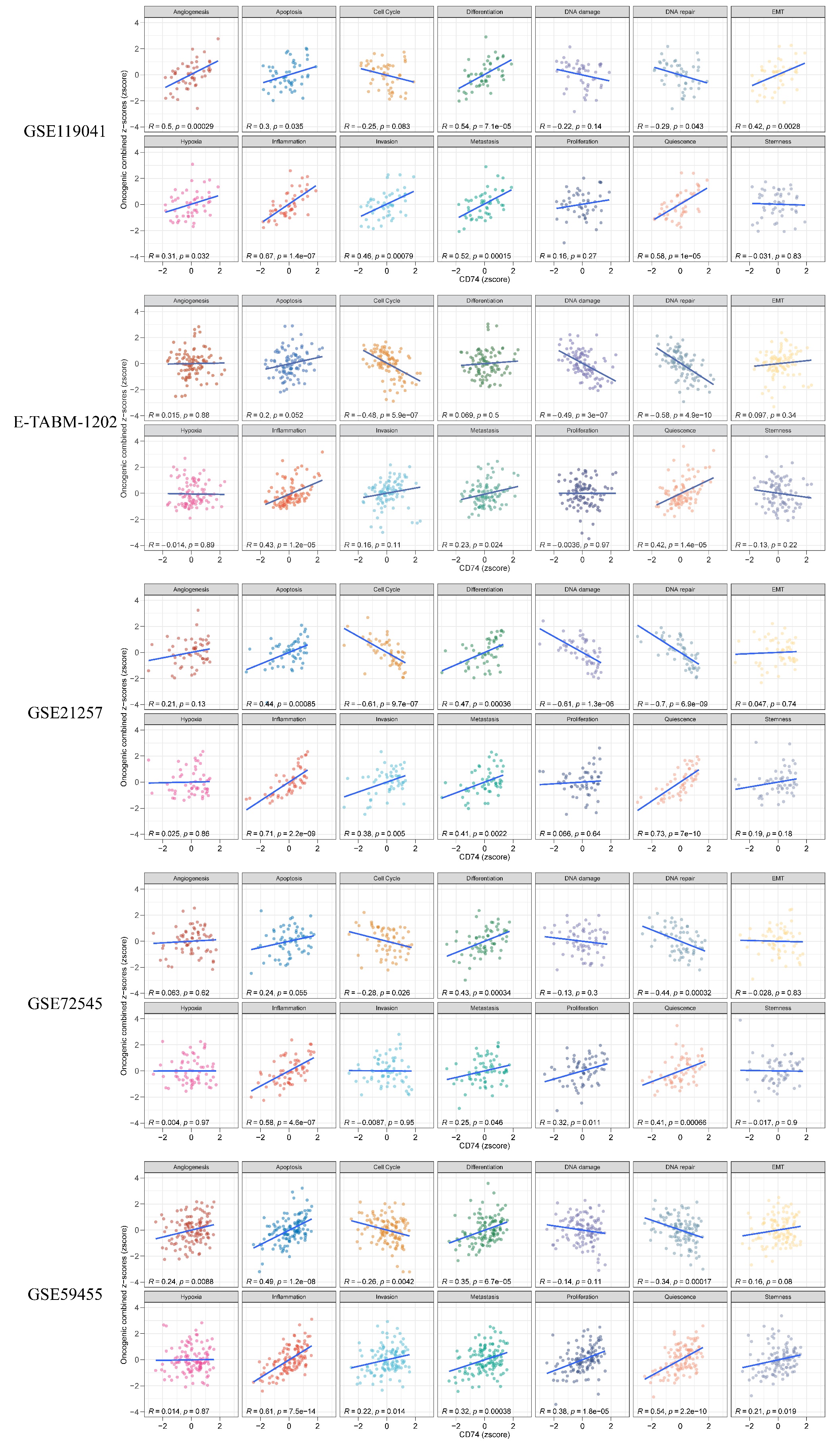


**Supplementary Figure 3**: Further external dataset validation of the prognostic model using Kaplan-Meier survival analysis. Dataset names are in brackets above plots. Red/blue curves represent high/low risk groups. Significant p-values (<0.05) from Log-rank tests are noted.


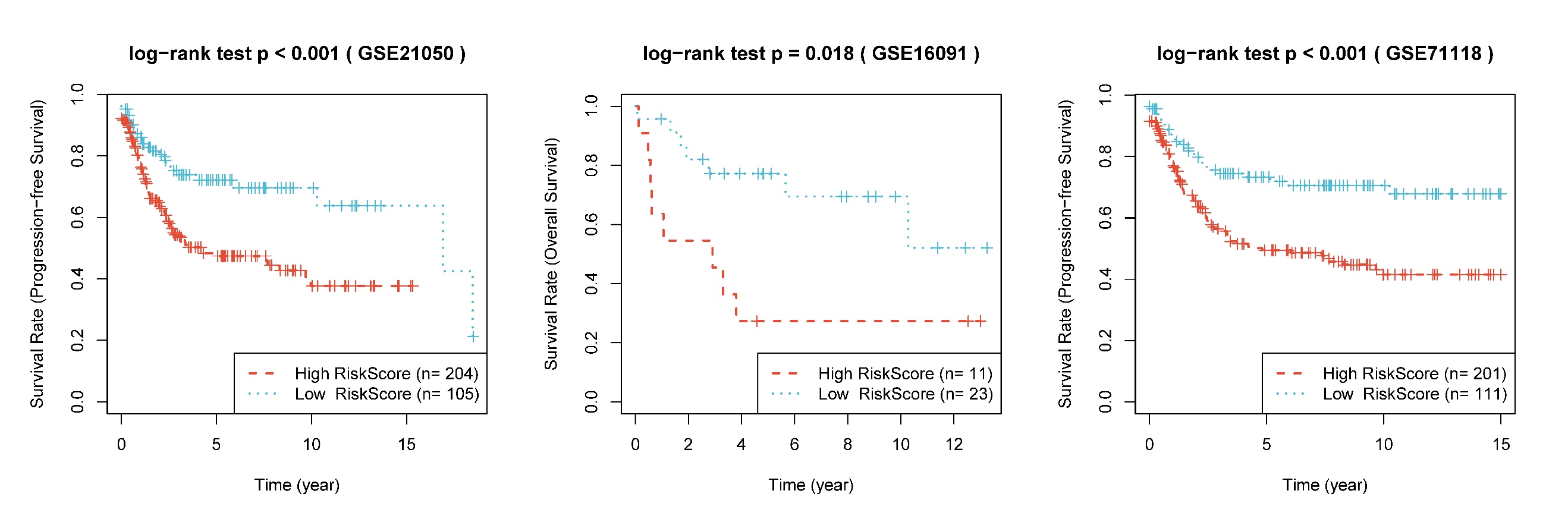


**Supplementary Figure 4**: Enhancing model robustness with a nomogram. The nomogram visually represents variable contributions to OS prediction, with total points estimating survival probabilities (A), and includes time-dependent ROC curves at 1, 3, and 5 years to illustrate model performance over time (B-D), as well as decision curve analysis (DCA) showing the net benefit of the nomogram compared to alternatives across threshold probabilities (E).


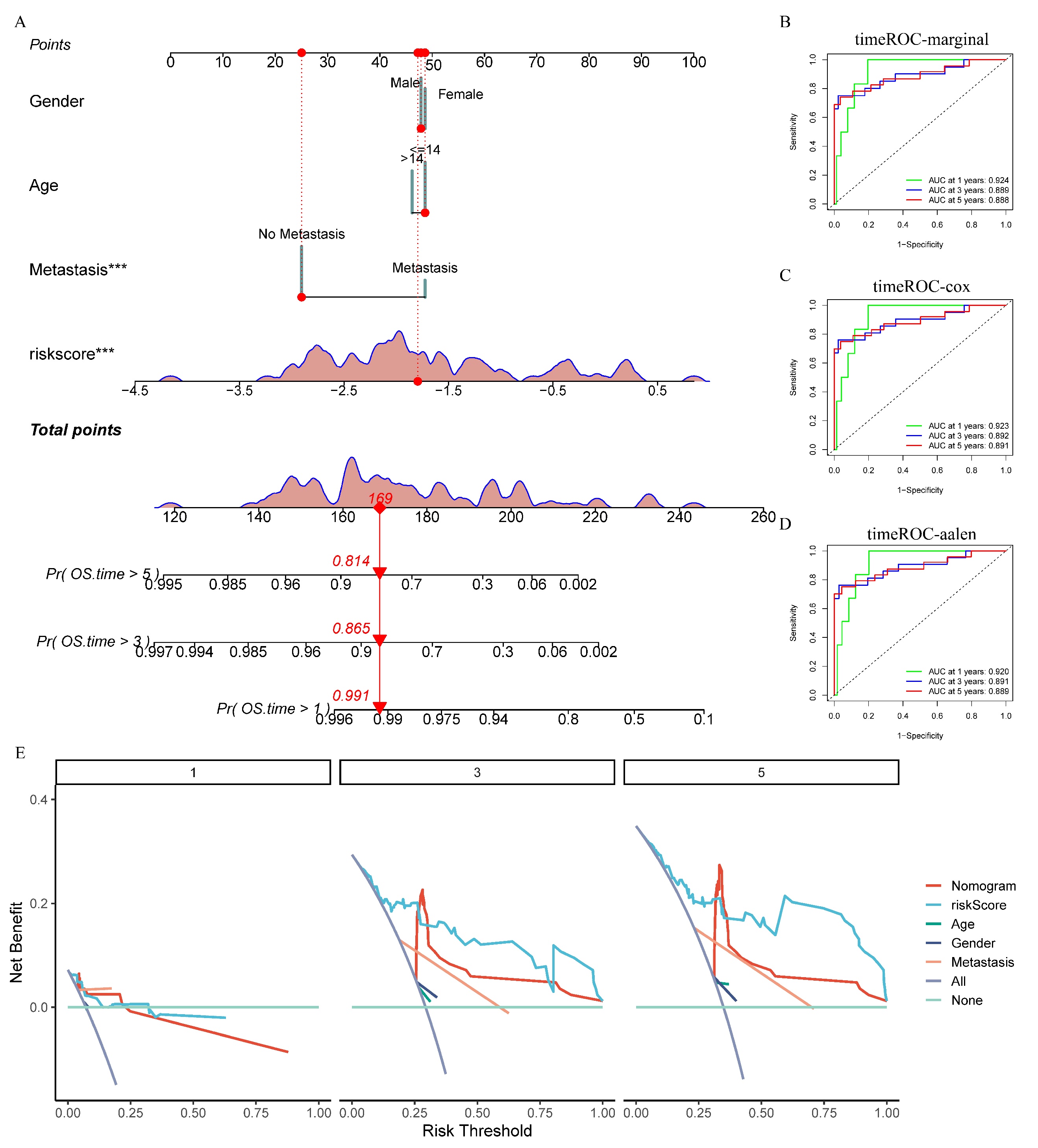


**Supplementary Figure 5**: Association between model and clinical variables. Risk score differences across clinical variables in the Target-OS dataset (A-C). Risk score differences across clinical variables in the GSE21257 dataset (D-F). Box plots show quartiles (Q1 and Q3) with whiskers extending to 1.5× IQR from Q1 and Q3


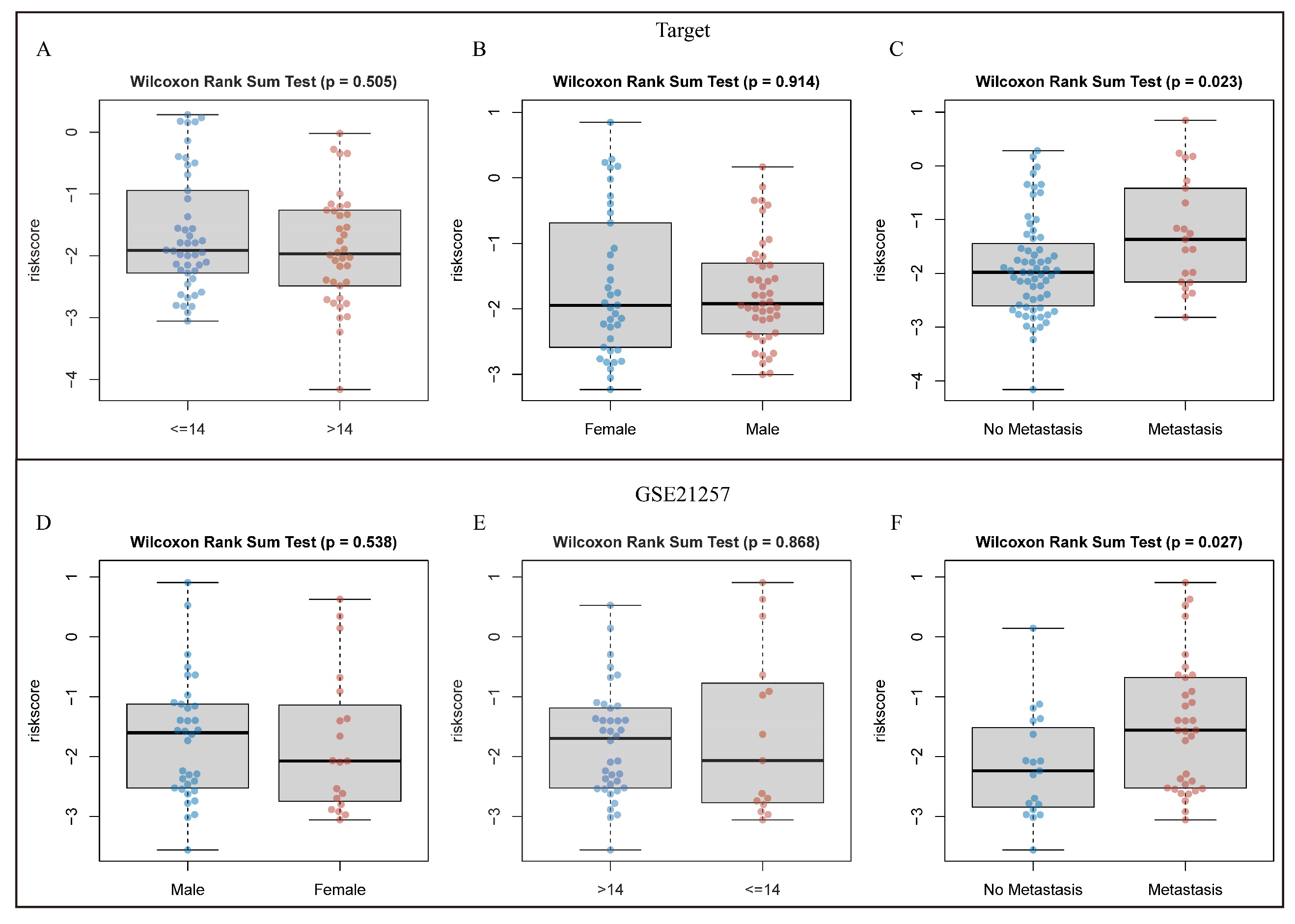

Supplement: Supplementary file 1 — Figure S1. Verification of the CAFs’ contribution to sarcoma oncogenic characteristics via the MIF‐CD74 signalling axis. Gene expression differences between tumour and normal tissues in cancer cohorts. Box plots show quartiles, with the line indicating the median. Wilcoxon Rank Sum Tests compare expressions between groups (A). The lollipop plot shows MIF expression (nTPM) across different tissues, with points representing gene expression in cancer cell lines (B). Scatter plot of potential interacting proteins centered on MIF, with coloured lines indicating subcellular localization evidence (C). Z‐score scatter plots of samples, coloured by subgroup, comparing MIF and fibroblast scores. Z‐scores ≤ 0 indicate low expression/scores, and > 0 indicate high (D–F). Kaplan–Meier survival analysis with log‐rank tests. Significant p‐values (< 0.05) are highlighted in grey‐backed tables (E–G). The bar plot summarises pathways significantly enriched in CD74 high/low expression groups, with colour indicating enrichment direction (H). Scatter plots show a correlation between functional status z‐scores and gene expression z‐scores, with colour indicating functional status type and R indicating Pearson correlation coefficient (I, J). Figure S2. Validation of scores of 14 tumour status across multiple external sarcoma datasets. Scatter plots show functional status z‐scores vs. gene expression z‐scores, coloured by functional status type, with R indicating Pearson correlation coefficient. Dataset names are labelled. Figure S3. Further external dataset validation of the prognostic model using Kaplan–Meier survival analysis. Dataset names are in brackets above plots. Red/blue curves represent high/low risk groups. Significant p‐values (< 0.05) from Log‐rank tests are noted. Figure S4. Enhancing model robustness with a nomogram. The nomogram visually represents variable contributions to OS prediction, with total points estimating survival probabilities (A), and includes time‐dependent R [file JCMM-29-e70424-s001.zip › figure.docx]
